# Pyeloplasty in Children with Ureteropelvic Junction Obstruction and Associated Kidney Anomalies: Can a Robotic Approach Make Surgery Easier?

**DOI:** 10.3390/children10091448

**Published:** 2023-08-25

**Authors:** Giovanni Cobellis, Edoardo Bindi

**Affiliations:** 1 Pediatric Surgery Unit, Salesi Children’s Hospital, Via F. Corridoni 11, 60123 Ancona, Italy; giovanni.cobellis@ospedaliriuniti.marche.it; 2 Department of Pediatric Surgery, Università Politecnica of Marche, 60121 Ancona, Italy

**Keywords:** robot-assisted pyeloplasty, pediatric surgery, renal anomalies, horseshoe kidney, nephrolithiasis

## Abstract

Background: Robot-assisted pyeloplasty is widely used in pediatric surgery because of its well-known advantages over open or laparoscopic surgery. The aim is to explore our experience and evaluate the achievements we have made. Methods: We evaluated patients undergoing robotic pyeloplasty from January 2016 to November 2021, including those who presented with a ureteropelvic junction obstruction associated with other anomalies of the kidney. The parameters examined were: age, weight, associated renal malformations, conversion rate, operative time, and intra- and postoperative complications. Results: Of 39 patients, 7 (20%) were included, of whom 5 (71%) were male and 2 (29%) were female. The mean age at surgery was 84 months (range 36–180 months), and the mean weight at surgery was 24.4 kg (range 11–40 kg). In five (71%) patients the ureteropelvic junction obstruction (UPJO) was left-sided and in two (29%) it was right-sided. In four (57%) cases, UPJO was associated with a horseshoe kidney, right-sided in one (25%) patient, and left-sided in the other three (75%). A 180° rotation of the kidney was present in one (14%) patient. Nephrolithiasis was present in two (29%) patients. The mean operative time was 160 min (range 140–240 min). The average bladder catheter dwell time was 1 day (range 2–3 days), while the average abdominal drainage dwell time was 2 days (range 2–4 days). The mean hospitalization time was 4 days (range 3–9 days). On average, after 45 days (range 30–65) the JJ ureteral stent was removed cystoscopically. No intraoperative complications were reported, while one case of persistent macrohematuria with anemia requiring blood transfusion occurred postoperatively. Conclusions: Ureteropelvic junction obstruction might be associated with other congenital urinary tract anomalies such as a duplicated collecting system, horseshoe kidney, or pelvic kidney. These kinds of malformations can complicate surgery and require more attention and accuracy from the surgeon. Our experience shows that, with regards to the robotic learning curve required for pyeloplasty, the treatment of the ureteropelvic junction in these situations does not present insurmountable difficulties nor is burdened by complications. The application of robot-assisted surgery in pediatric urology makes difficult pyeloplasties easier.

## 1. Introduction

Ureteropelvic junction obstruction (UPJO), with an incidence of 1:1250 births, represents one of the most frequent urinary tract anomalies in the pediatric population. UPJO can be associated with vesicoureteral reflux in 12% of cases [1]. UPJO can be presented in association with other congenital anomalies of the urinary tract such as a duplication of the collecting system, horseshoe kidney (HSK), or pelvic kidney (PK) [2]. Moreover, in 2.1% of patients, UPJO is complicated by the coexistence of nephrolithiasis [3].

Pyeloplasty is the gold standard for the treatment of UPJO and since 1995, the year of the first laparoscopic pyeloplasty in pediatric patients [4], it has been established with a minimally invasive approach [5]. In recent years, the development of robotic surgery applications in pediatric surgery has raised the bar further, and currently, robot-assisted pyeloplasty is considered a safe and effective approach for the treatment of this pathology [6,7,8]. The advantages of robotic surgery over laparoscopic surgery are well known, but in cases of UPJO complicated by the association of other malformations or renal pathologies, the usefulness of the robot-assisted approach is even more readily apparent.

In adults, there are case reports which have addressed endoscopic surgery, laparoscopic surgery, and recently, robotic approaches to UPJO associated with congenital renal anomalies such as ectopic pelvic kidneys [9,10]. To our knowledge, there are only limited data on the surgical outcome of UPJO and kidney anomalies in children. In a work from 2018 [2], the authors presented their experience with transperitoneal laparoscopic pyeloplasty in children with UPJO associated with complex anatomical anomalies.

In this work, we aimed to describe our experience with robot-assisted pyeloplasty for this kind of situation. Our purpose is to demonstrate how the robot can make surgical procedures easier, even in difficult pyeloplasties.

## 2. Materials and Methods

This study was carried out at the Department of Pediatric Surgery at the Salesi Children’s Hospital, in Ancona. We performed a retrospective study on pediatric patients undergoing pyeloplasty with the robot-assisted technique from November 2016 to November 2021. The Si and Xi Da Vinci robotic systems were used in all cases. We considered the patients who presented ureteropelvic junction obstruction (UPJO) in association with other renal malformations or diseases.

We collected data on the characteristics of the patients, the diseases we treated, the surgical procedures performed, and any complications.

The inclusion criteria were:

- Patients operated by robot-assisted technique;
- Patients who presented ureteropelvic junction obstruction (UPJO) in association with other renal malformations or diseases.
- The exclusion criteria were:
- Patients operated with other surgical approaches (open surgery, retroperitoneoscopy, laparoscopy);
- Patients with UPJO and no other renal anomalies.

Since this study did not involve any direct experimental intervention on patients but was based only on a retrospective collection of patient data, we did not need IRB approval.

The hypothesis we wanted to evaluate in this study was that we believe the robot-assisted approach can improve and facilitate surgery in patients with complex anatomical situations.

The primary outcome evaluated was the conversion rate and the secondary outcomes were the rate of recurrence and intraoperative and postoperative complications (Table 1).

### Robot-Assisted Pyeloplasty

Experience in robot-assisted surgery developed in recent years has enabled us to implement operative protocols to standardize the patient’s surgical pathway from the moment before entering the room to postoperative care.

The robotic trocars used were 3: one of 12 mm for the optic (inserted at the umbilical level with an open technique) and two of 8 mm. Another laparoscopic trocar of 5 mm, for the assistant surgeon was positioned at the table. The robotic trocars were placed on the same line at nearly 10 cm of distance from each other.

The patient was lying down on the operating table on the left side at a 60-degree angle operating surface. The patient was placed on the edge of the operating table opposite the Da Vinci robot trolley.

The pyeloplasty procedure followed the classic steps described by the Anderson–Hynes technique.

## 3. Results

During the studied period, we performed 39 robot-assisted pyeloplasty procedures. Among these patients, we selected those who had UPJO associated with kidney anomalies. Seven (20%) patients were included, of whom five (71%) were male and two (29%) were female. The mean age at surgery was 84 months (range 36–180 months), and the mean weight at surgery was 24.4 kg (range 11–40 kg). Patient demographics are shown in Table 2.

In five (71%) patients the ureteropelvic junction obstruction (UPJO) was left-sided and in two (29%) it was right-sided. In four (57%) cases, UPJO was associated with a horseshoe kidney, right-sided in on (25%) patient and left-sided in the other three (75%). A 180° rotation of the kidney was present in one (14%) patient. Nephrolithiasis was present in two (29%) patients.

In all patients, the first identification of hydronephrosis was by ultrasonography. In two (29%) patients the diagnosis of hydronephrosis was made by prenatal ultrasonography, while in the others ultrasonography was performed postnatally. In two (29%) patients hydronephrosis was detected during an ultrasound performed for other reasons, while in three (42%) cases, the patient had presented with recurrent abdominal pain.

**Figure 1:**
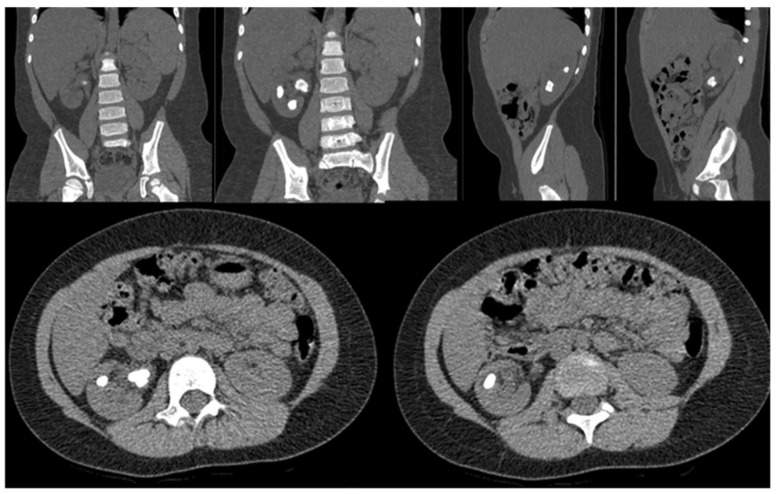
Abdominal CT scan showing right nephrolithiasis in a kidney affected by right UPJO.

In the two patients in whom the presence of renal stones had been detected by ultrasonography, an abdominal CT (Figure 1) was performed to better define nephrolithiasis.

Following our center’s protocol, all patients underwent renal scintigraphy with MAG3 to detect the presence of ureteropelvic junction obstruction and assess renal function. Renal function of the affected side was preserved in all patients except two (29%) who had split renal function of the pathologic kidney of 20.6% and 13.1%, respectively. In these two cases, however, we retained the indication to perform pyeloplasty, as the indication for nephrectomy is placed for renal function below 10%.

**Figure 2:**
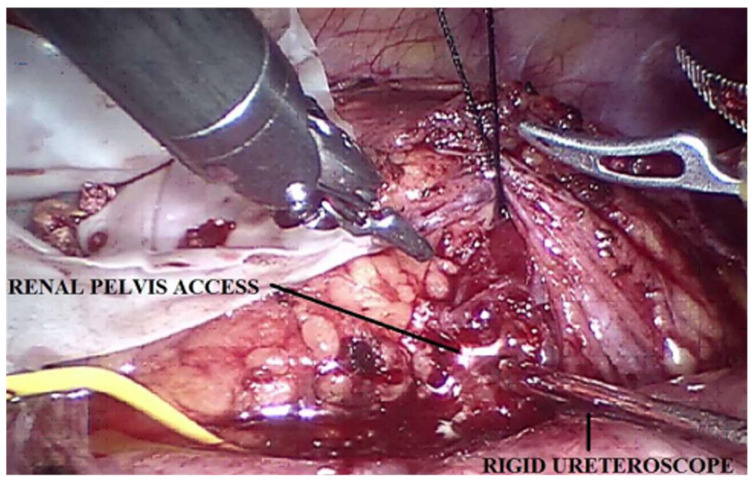
Robot-assisted approach to a patient affected by UPJO associated with nephrolithiasis. This technique permits us to easily perform a pyeloplasty and to clear the renal stones at the same time.

In all patients, a robot-assisted pyeloplasty was performed. In the two (29%) patients in whom nephrolithiasis was present, stone clearance was performed. In one case, the stones were simply removed by widening the renal pelvis incision and extracting them from the lower calyces (Figure 2). In the second case, once the renal pelvis was opened, a rigid ureteroscope (Ch 10) was inserted through the accessory laparoscopic port of 5 mm in the pelvis; nevertheless, the calyces that resulted were not easy to reach. Through the same trocar access, a flexible renoscopy was performed using a digital disposable flexible instrument. Without further increasing the pressure, irrigation of saline was used to permit viewing of the pelvis and calyces; the second robotic arm made it possible to maintain suction. A second stone was later identified, which was removed with a Dormia basket. Since the fact that the stones located in the medium and inferior calyx were larger than the circumference of the neck of the calyx, the extraction with the basket was not feasible. We performed lithotripsy with a Holmium laser through a 0.27 mm fiber. The mean operative time was 160 min (range 140–240 min). The average bladder catheter dwell time was 2 days (range 2–3 days), while the average abdominal drainage dwell time was 2 days (range 2–4 days). The mean hospitalization time was 4 days (range 3–9 days). On average, after 45 days (range 30–65) the JJ ureteral stent was removed cystoscopically.

No intraoperative complications were reported, while one case of persistent macrohematuria with anemia requiring blood transfusion occurred postoperatively. In this case, we explained macrohematuria as a suspected mucosal injury of the ureter by the stent. We ruled out the possibility of vascular injury with a Doppler ultrasound examination. The bleeding resolved spontaneously within 12 h. We performed a check of hemoglobin, which had dropped 1.5 g/dL (from 9.5 g/dL to 8 g/dL). The patient retained stable hemodynamic parameters, but we still preferred to perform a transfusion of concentrated hematins.

There were no conversions in our series.

The patients were then followed in a postoperative follow-up, which included ultrasound at 1 and 6 months after surgery. Renal scintigraphy with MAG3 was performed 1 year later. The average follow-up period was 1.5 years; patients presented with good condition, no episodes of urinary infections or abdominal pain, and no recurrences.

## 4. Discussion

When it comes to minimally invasive techniques, the advantages that this approach has brought to surgery are now well-known [11]. The advantages of minimally invasive surgery concerning traditional open surgery consist of less surgical trauma and loss of blood, fewer postoperative complications, less postoperative pain, shorter hospitalization time, and improved outcomes regarding the cosmetic result. In addition, robotic instruments, thanks to their technology and the possibility of 360 degrees of rotation, can improve precision and accuracy, by eliminating tremors due to the operator’s movements. These characteristics extend the use of minimally invasive surgery in order to be applied also in more difficult procedures that would otherwise need open surgery. Robotic procedures are safe and appropriate for pediatric interventions that often need accurate dissection and suturing in small anatomical spaces.

Nonetheless, the expansion of robotic surgery in pediatric settings has faced several limitations. One of these difficulties has been the smaller anatomical working space of pediatric patients, which can reduce or obstruct the movements of robotic instruments. The improvements in the technological features of the instruments have partially resolved these difficulties, but the selection of patients is still necessary and mandatory for the safe and successful use of robotic technology in a pediatric setting.

Even though in pediatric surgery minimally invasive techniques came later and developed more slowly, the laparoscopic, thoracoscopic, and/or robot-assisted approach has become the most widely used choice. In our center, minimally invasive surgery has found wide application for many years now. Starting from a laparoscopic learning curve in the abdominal field [12], we have tried to develop the technique more and more with good results in urological treatment as well [13,14]. In urology in particular, robot-assisted surgery has brought the greatest benefits and the number of procedures performed has increased over time, as has the learning curve for pediatric surgeons [7,15]. Among these, pyeloplasty was one of the first procedures in which robotics became established as an effective technique and currently, robot-assisted laparoscopic pyeloplasty (RALP) represents one of the most frequently performed procedures, even in pediatric settings [16]. This technique, compared with laparoscopic pyeloplasty (LP), has demonstrated the same success rate and complications, but with a statistically significantly shorter mean time for anastomosis in robotic pyeloplasty compared to laparoscopic pyeloplasty [17,18,19]. In addition to the shorter time spent performing ureteropelvic anastomosis, it should be noted that the movements of robotic instruments allow this procedure to be performed with greater precision than laparoscopy and with less fatigue for the surgeon.

One of the main indications of robotics is its use for surgical procedures in which the reconstructive component is predominant. Therefore, in this paper, we wanted to analyze our experience with RALP performed in complex cases of ureteropelvic junction obstruction.

UPJO may be associated with other malformations of the urinary system or other kidney diseases, which may complicate its treatment. A duplicated collecting system, horseshoe kidney (HSK), or pelvic kidney (PK) are conditions that could require more caution during surgery.

The horseshoe kidney (HSK) is a well-described renal fusion and anomaly of rotation, with an incidence of 1 in every 500 individuals. Despite the fact that horseshoe kidney amounts to only about 0.25% of the population, this kind of congenital anomaly is a very common defect of fusion of the kidneys. The description of this malformation was performed in 1522 by da Carpi. Abnormalities in the position, rotation, and vasculature of the kidney are the main characteristics of a horseshoe kidney [20]. HSKs have been described as formed of functioning renal parenchyma fused with two ureters that never cross each other throughout their entire course from hilum to bladder. The isthmus is usually positioned in the midline or laterally with the result of an asymmetric horseshoe kidney. It is made of renal parenchyma in more than 75% of cases with the remainder being made of a fibrous tissue that connects the two renal units. In more than 85% of patients, fusion occurs at the level of the lower pole but cases have also been reported of fusion occurring at the upper pole in a small percentage of people. Horseshoe kidneys can be asymptomatic, so their identification can be performed incidentally. The symptoms can be those of other common renal diseases, with patients presenting complaints of abdominal pain or symptoms of a urinary tract infection. Three characteristics can differentiate horseshoe kidneys from the normal renal anatomical situation: position, orientation, and vasculature. The horseshoe kidney may be located medially in the abdomen, down from the inferior mesenteric vein at the level of L3, or it may be located at the level of the lower quadrants of the abdomen or even in a pelvic position. The anomaly of rotation of the horseshoe kidney, caused by the conformation of the isthmus, can result in a different pathway of the ureters, which can run either anteriorly or posteriorly to the renal surface. This course may result in ureteral kinking with obstruction of urinary outflow. HSK can present variations in the origin and number of renal arteries and veins. This problem is due to where ascent has stopped during embryological development. Nevertheless, the normal intra-renal vascular segmental pattern remains, and the interruption of any of these branches can result in ischemia of the kidney or necrosis due to the paucity of anastomotic circles. Treatment of joint stenosis in these situations, given the low frequency of cases and anatomical complexity, has traditionally been approached by performing open pyeloplasty according to the Anderson–Hynes technique. In 2012, a large retrospective study [21] reviewed 680 pediatric patients treated for UPJO in 15 years. Of these, 43 presented an association with renal malformation. The entire cohort of patients underwent open pyeloplasty with a postoperative success rate of nearly 52%. Four patients presented with recurrence during follow-up and required redo pyeloplasty, while three underwent nephrectomy for progressive loss of renal function. The same authors conclude that the results are conditioned by the complex renal anatomy, which makes dissection and joint reconstruction even more difficult. In addition, the open approach carries with it all the disadvantages that are now known and have long since been overtaken by minimally invasive surgery in this type of surgery. The laparoscopic approach has certainly revolutionized the treatment of ureteropelvic junction obstruction, but it remains a procedure with a more difficult learning curve compared with other laparoscopic procedures. The presence, then, of a malformation adds further difficulties, consisting mainly of the abnormal positioning of the junction that must be dissected and then reconstructed. Aberrant lower pole vessels, the caudal position of the kidney, and the renal isthmus represent the most common technical challenges of pyeloplasty in this population. There are not many papers in the literature describing laparoscopic pyeloplasty in the horseshoe kidney, and few of these analyze an exclusively pediatric population. A 2018 study [2] analyzes a series of 11 pediatric patients undergoing laparoscopic pyeloplasty in whom a renal malformation was present. Of these, six patients presented with a horseshoe kidney. The authors report good results in terms of resolution of the stenosis in the absence of major complications. No statistically significant difference is reported in terms of operative time compared with pyeloplasty performed in normal kidneys. They point out that, in addition to the similar operative time, the laparoscopic approach presents a lower morbidity relative to the open approach and an extremely good view of the anomalies of the anatomy, like vessels and ureteral position. Nevertheless, pyeloplasties and other reconstructive laparoscopic procedures required a long and difficult learning curve that cannot be reached in an easy way by centers with a low volume of activity, especially when they have to manage complex and rare cases. Another work from 2013 [22] presents a case history of nine patients, including five adults and four pediatric patients, who had UPJO on horseshoe kidneys. The pediatric patients were treated with a robotic approach, but only in two of them was a pyeloplasty performed. The authors report similar results compared with the laparoscopically operated adult counterpart, highlighting a shorter postoperative hospital stay as the difference.

In our work, we confirm how the surgical complexity of these cases is significantly decreased by the robotic approach. Robot-assisted pyeloplasty demonstrated to have a shorter operative time and a dramatically shorter learning curve than laparoscopic pyeloplasty. In addition, the dexterity of robotic instruments makes it possible to safely perform the dissection and the reconstruction of the pelvis.

**Figure 3:**
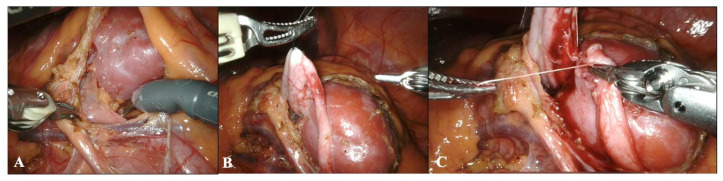
A case of a patient with a horseshoe kidney in which the renal pelvis is hidden (**A**). The great freedom of movement of the robotic instruments allows them to reach the pelvis (**B**) and perform pyeloplasty with ease (**C**).

The more problematic elements represented by the more hidden location of the pelvis and the difficulty in locating the course of the ureter and renal vessels are solved by the dexterity of robotic instruments and 3D vision (Figure 3). These distinctive aspects of robot-assisted surgery are found to be even more critical in the second stage of the procedure, that of joint reconstruction, where they facilitate the surgeon’s work and make the anastomosis safer.

In our results, the operative time was similar to that of other robot-assisted procedures, with the same postoperative hospital stay. Therefore, such an operation, which in laparoscopy would have longer surgical and anesthesiological times, with the robot results in a duration comparable to that of a standard pyeloplasty. In addition, in our experience, the learning curve in robotic surgery developed over the years has facilitated the approach to these more surgically complicated situations.

Another condition that can complicate a pyeloplasty is nephrolithiasis. Kidney stones in children have been managed in the past by general urologists, who applied the standards and protocols used for adult patients. As currently can be well understood, the problem is that clinical presentation, etiology, and treatment are significantly different between childhood and adulthood. In managing this kind of disease, there are specific characteristics in pediatric patients, such as the presence of underlying metabolic anomalies, which can lead to the risk of the development of progressive renal dysfunction without an adequate diagnosis and treatment. Nowadays it is mandatory to make a diagnosis of an anomaly in metabolism in children with nephrolithiasis, to establish personalized therapy. Over the last 20 years, the surgical approach in pediatric patients has changed in a significant way: the traditional open surgical approach has been almost completely replaced by minimally invasive surgery. This condition, which once seemed to be the sole concern of the urologist, is currently a growing problem among kids, constituting a disease entity even for the pediatric surgeon. In young patients, this can result in recurrent pyelonephritis, an increase in blood pressure, and progressive dysfunction of the kidney without adequate therapies [23]. In UPJO, the obstruction and the consequent stasis of urine can increase the percentage of renal stone formation by more than 20%, as reported by several articles in the international literature [24]. The best treatment for this kind of patient presents several problems and difficulties. The goal of the surgeons is to reach a stone-free status with a few minimally invasive interventions and with no intra-operative or postoperative complications. Recent years have seen several procedures, like retrograde intrarenal surgery, percutaneous nephrolithotomy (PCNL), endoscopic combined intrarenal surgery, and extracorporeal shockwave lithotripsy (ESWL); these procedures are associated with varying rates of residual or recurrent stones. Several articles described percutaneous nephrolithotomy as the first treatment for patients with stones larger than 1 cm thanks to its low morbidity and extensive stone-free rates [25,26]. Since 2005, it has been shown that in these patients, the treatment that offered better results combined pyeloplasty with intraoperative stone clearance [27]. In this sense, especially initially, the choice of surgical approach fell on laparoscopy. Experiences in this regard, however, have shown that this is a surgery that requires high laparoscopic skills that are not easy to learn, resulting in a longer operative time without a clear improvement in postoperative results [28]. In adults, robotic surgery is an approach for operating on UPJO that has proved satisfactory results. With this procedure, it is also possible to remove calculi from the pelvis or from the calyx. There are well-known advantages related to the robotic technique that makes the reconstruction of the pelvic junction easier, such as the presence of a wrist in each instrument, seven degrees of freedom, extraordinary visualization, no tremors in the movements, precise suture placement, and watertight closure of the collecting system [29]. The robotic approach in pediatric settings is beginning to be reported in the literature, but the series remains limited. The data, although initial, show that pyeloplasty and stone clearance performed robotically have, even in these patients, good results in terms of resolution of pathology, low risk of recurrence, and safety [30]. A review in 2022 highlighted that robot-assisted laparoscopic pyeloplasty (RALP) has been demonstrated to be an innovative and effective surgical treatment for select cases of children affected by urolithiasis. At present, data in the literature are still limited, but pediatric patients who present an obstruction of the ureteropelvic junction in association with nephrolithiasis appear to become a specific group with a strong indication for the robot-assisted intervention. Combining a flexible ureteroscope with robot-assisted approach can correct UPJO and remove renal calculi at the same time. This kind of approach can avoid the need for more interventions and it can reduce the number of anesthesiological procedures with the potential advantage of lowering the morbidity associated with multiple access routes to the kidneys [31,32,33,34]. Our experience, although limited, was able to show similar results, which proved encouraging both because the operative time was not significantly longer than that of standard pyeloplasty and because the operative act, thanks to robotic technology, did not prove difficult to perform.

## 5. Conclusions

Talking about the technical aspects of this kind of surgery, laparoscopic procedure is considered an advanced intervention among the MIS approaches, as it needs a high level of skill, related to suturing and knotting in limited spaces [35,36,37]. Conversely, robot-assisted surgery permits one to limit some of these technical difficulties inherent in laparoscopy, despite the need for a long learning curve. Moreover, the learning curve for suture placement in robotic surgery is shorter compared to laparoscopic surgery. The characteristics of the robotic system permit the passage from open to robot-assisted laparoscopic pyeloplasty without previous experience in conventional laparoscopy [38,39,40,41]. To accurately and safely perform surgery using a laparoscopic technique, it is necessary to have a high level of skill for difficult procedures that require time for reconstruction, such as laparoscopic intervention for UPJO. On the other hand, robotic surgery has great and efficient potential for making minimally invasive surgery more accessible and easier for these types of procedures. For these reasons, robotic technology has contributed to shortening and to making it less difficult to develop the learning curve. We can say that robotic surgery acts as a bridge between open and minimally invasive surgery. Consequently, whereas laparoscopy is performed only in some select surgical centers with a high volume of minimally invasive activity on pediatric patients, robot-assisted pyeloplasty is now performed in every pediatric surgical center with a robotic program [42,43,44,45,46].

The possibility of having, at our center, the DaVinci robotic system, allowed us in a short time to be able to perform an adequate number of pyeloplasties, such that a good learning curve was developed. This ensured that we had a base from which we could start to tackle even more technically difficult cases that we had little experience with, given also the rarity of the associated conditions. The development of a satisfying learning curve and the advantages provided by robotic technology allowed us to treat these “difficult” pyeloplasties safely and with satisfactory results. In our opinion, therefore, we can say that robotic approach represents a frontier to be explored and developed for the future, as it allows us to overcome even the most complicated situations faced by the surgeon.

## Figures and Tables

**Table 1:** Hypothesis and outcomes of the study.

**Hypothesis of the study**	The robot-assisted technique facilitates surgery even in complex anatomical situations.
**Primary outcome**	Conversion rate
**Secondary outcomes**	- Recurrence rate - Intraoperative complications - Post-operative complications

**Table 2:** Study population data.

Patients	7
Males/Females	5 (71%)/2 (29%)
Side of UPJO: Right/Left	2 (29%)/5 (71%)
Associated renal anomalies	- 4 (57%) horseshoe kidney - 1 (14%) 180° rotation of the kidney - 2 (29%) nephrolithiasis
Mean age at surgery	84 months (range 36–180 months)
Mean weight at surgery	24.4 kg (range 11–40 kg)
Mean operative time	160 min (range 140–240 min)
Mean hospital stay	4 days (range 3–9 days)
Intraoperative complications	0
Postoperative complications	1 (14%) macrohematuria
Conversions	0

## Data Availability

Not applicable.
